# RightPath: a model of community-based musculoskeletal care for children

**DOI:** 10.1093/rap/rkaa057

**Published:** 2020-10-16

**Authors:** Nicola Smith, Victoria Mercer, Jill Firth, Sharmila Jandial, Katharine Kinsey, Helen Light, Alan Nye, Tim Rapley, Helen E Foster

**Affiliations:** Population Health Sciences Institute, Newcastle University, Newcastle Upon Tyne; Population Health Sciences Institute, Newcastle University, Newcastle Upon Tyne; Physiotherapy, South Tyneside and Sunderland NHS Foundation Trust, South Shields; Pennine MSK Partnership, Oldham; Population Health Sciences Institute, Newcastle University, Newcastle Upon Tyne; Paediatric Rheumatology, Great North Children’s Hospital, Newcastle upon Tyne; Pennine MSK Partnership, Oldham; Pennine MSK Partnership, Oldham; Pennine MSK Partnership, Oldham; Department of Social Work, Education and Community Wellbeing, Northumbria University, Newcastle Upon Tyne, UK; Population Health Sciences Institute, Newcastle University, Newcastle Upon Tyne; Paediatric Rheumatology, Great North Children’s Hospital, Newcastle upon Tyne

**Keywords:** child health, primary health care, qualitative research, patient perspectives, triage, service development

## Abstract

**Objectives:**

Musculoskeletal (MSK) presentations are common (reported prevalence of one in eight children) and a frequent cause of consultations (6% of 7-year-olds in a cohort study from the UK). Many causes are self-limiting or raised as concerns about normal development (so-called normal variants). We aimed to describe a new model of care to identify children who might be managed in the community by paediatric physiotherapists and/or podiatrists rather than referral to hospital specialist services.

**Methods:**

Using mixed methods, we tested the feasibility, acceptability and transferability of the model in two UK sites. Evaluation included patient flow, referral times, diagnosis and feedback (using questionnaires, focus groups and interviews).

**Results:**

All general practitioner referrals for MSK presentations (in individuals <16 years of age) were triaged by nurses or allied health professionals using a triage guide; ∼25% of all MSK referrals were triaged to be managed by community-based paediatric physiotherapists/podiatrists, and most (67%) had a diagnosis of normal variants. Families reported high satisfaction, with no complaints or requests for onward specialist referral. No children re-presented to the triage service or with serious MSK pathology to hospital specialist services in the subsequent 6 months after triage. Triagers reported paediatric experience to be important in triage decision-making and case-based learning to be the preferred training format.

**Conclusion:**

The triage model is acceptable, feasible and transferable to enable appropriate care in the community for a proportion of children with MSK complaints. This is a multi-professional model of better working together between primary community and specialist providers.

**Key messages**

- RightPath is an innovative model of care with improved cross-boundary working.
- The RightPath model aims to reduce variation in care and provide clarity of care pathways.
- Effective triage in the community by allied health professionals facilitates better utilization of resources.

## Introduction

Musculoskeletal (MSK) presentations in children and young people are common (reported prevalence of one in eight children [1]) and a frequent cause of consultations increasing with age (6% of 7-year-olds to 16% of 22-year-olds in a cohort study from the UK) [2]. General practitioners (GPs) are often the first health-care professionals (HCPs) to assess children and play a crucial role in deciding on specialist hospital referral (or not) and initiating care pathways [3, 4].

Many causes of MSK presentations in primary care are self-limiting (e.g. related to viral illness or mild trauma) or are raised as concerns about normal development (e.g. flat feet or bow legs; so-called normal variants) [1, 2, 5]. However, some causes include serious MSK red-flag conditions (e.g. bone and joint infection, bone malignancy or non-accidental injury) or potentially disabling pathology (e.g. orthopaedic hip conditions, rheumatic diseases or neuromuscular problems). The management of such conditions is a priority for UK National Health Service (NHS) hospital-based specialist services (https://www.england.nhs.uk/commissioning/spec-services/), especially given that delay, which is well reported, impacts adversely on clinical outcomes [3, 6–12]. 

Our study was based on the premise that some children with MSK presentations could be assessed and managed in the community rather than being referred to a hospital specialist service. We developed a new model of care, based on an established adult MSK triage service (https://www.pmskp.org) and within existing frameworks of UK primary and community care NHS clinical pathways [13]. This new model, which we have called RightPath, aims to identify children appropriate for assessment and management by paediatric physiotherapy or podiatry within the community and also to identify those with suspected MSK pathology for referral to hospital specialist services. In this paper, we describe the development, implementation and initial evaluation of the RightPath model. 

## Methods

**Fig. 1:**
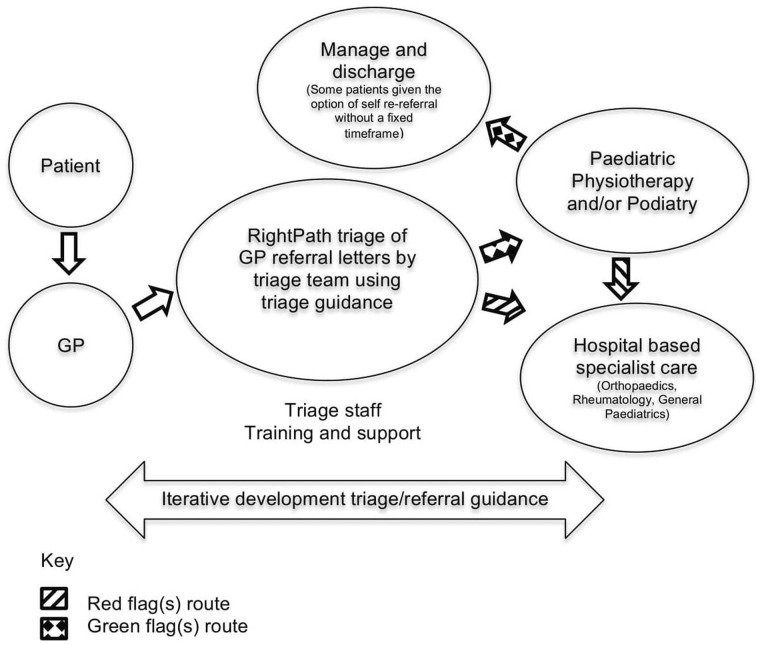
The RightPath model GP: general practitioner.

An overview of RightPath is given (Fig. 1). The study was based in two sites to test transferability, with a ∼3 month interval between starting the study at the second site to allow iterative modification based on feedback.

Site 1 was Oldham, UK in North West England and the site of the existing adult MSK service (https://www.pmskp.org) with an e-portal receiving all referrals from 43 GP practices and catchment total population of ∼235,000 [14]. The RightPath triage process started at the point of triaging GP referral letters received via an e-portal.

Site 2 was South Tyneside and Sunderland NHS Foundation Trust, North East England, UK, with an existing paediatric gait variant community-based service led by paediatric physiotherapists and with a general paediatrics department within the same NHS Trust, with both receiving direct referrals from 21 GP practices and serving a total population of 150,300 [15]. The RightPath triage process started at the point of receiving GP referral letters at the NHS Trust, either in general paediatrics or in paediatric physiotherapy.

Both sites had pre-existing onward referral access to a regional NHS Children’s hospital with established specialist paediatric rheumatology and paediatric orthopaedic services. Neither study site had pre-existing triage guidance for children with MSK presentations.

Stakeholder consultation events at each site before the start of the study engaged local communities and aired concerns about potential de-stabilization of services; attendees included primary, community and hospital specialist care representatives and members of the British Society for Paediatric and Adolescent Rheumatology Parents Group. A report of the discussions was sent to all primary care providers in the respective areas and followed up with regular e-Newsletters to describe progress of the study.

### Triage guidance

The triage guide was developed by the project team, who had varied paediatric MSK expertise. The triage guide focused on common MSK lower limb scenarios (e.g. flat feet, knee pain, foot pain) based on audits of anonymized referral data from each site before the start of the project. The triage guide aimed to identify normal variants and other non-serious MSK lower limb conditions (such as anterior knee pain and sports injuries) that could be managed by community-based providers (i.e. paediatric physiotherapists/podiatrists). We anticipated that subtle forms of MSK conditions, such as inflammatory arthritis without a sick child presentation, could potentially come through RightPath; the triage guide therefore included green flags (reassurance indicators) and red flags (indicators of concern), with further referral guidance to rheumatology, orthopaedics, neurodisability or general paediatrics. The triage guidance is available (www.RightPath.solutions).

### Triage teams

The triage teams comprised HCPs who triaged all GP referral letters for children (≤16 years of age) that included any MSK complaint(s). Triagers used the triage guide to identify those deemed appropriate for a community-based appointment with a paediatric physiotherapist and/or podiatrist. The remainder continued with their referral to secondary care. Triage decisions using the triage guide were based on the content of the GP referral letter; we had no direct interaction with the GP, and there was no proforma or influence over the GP referral decision. Triagers were encouraged to have a low threshold for onward referral to hospital specialist services and to collate personal reflections in a weekly log, noting challenges, concerns and actions taken. The role of paediatric experience in the application of the triage guide was considered likely to be important and was included as part of our study planning and explored further in the evaluation (confidence logs and qualitative work).

Triage staff at site 1 had access to advice from a GP with a specialist interest in rheumatology and a consultant adult rheumatologist with experience of adolescent rheumatology (both project team members at site 1) for informal *ad hoc* advice and case-based practice group discussions based on real-life data, logbooks and feedback. All triagers had access to PMM (paediatric musculoskeletal matters; www.pmmonline.org) as a free online learning resource [16].

### Evaluation

Evaluation included patient demographics, triage decisions, referral times and eventual diagnosis, where available. To assess safety, ethical permission included notification by the respective NHS Trusts of any patients who had been triaged and who subsequently presented with a diagnostic code reflecting a red-flag condition (namely septic arthritis, osteomyelitis or malignancy) ≤6 months after termination of the study at each site; the assumption being that serious illnesses would re-present within this time frame. Furthermore, children managed in the community also had the option of self re-referral back to the paediatric physiotherapist or podiatrist.

For those children triaged to be assessed by community providers, evaluation included feedback from parents using the validated Friends and Family questionnaire [17] to explore satisfaction and Collaborate [18] as a measure of shared decision-making; both were completed by consented parents immediately after consultations. Parents at both sites were also invited to take part in a telephone interview after the consultation.

Qualitative methods explored experiences from triagers and community providers, using focus groups at three time points during the study and one-to-one interviews to explore emerging themes. Insights into training needs were informed by weekly logs (using a five-point Likert scale about ease of triage decisions), focus groups and interviews.

Consent was obtained from all participants in the focus groups, interviews and weekly logs. All patient and participant information was anonymized. Focus groups and interviews were audio-recorded and transcripts anonymized and analysed following standard procedures for qualitative analysis, including open and focused coding, constant comparison and deviant case analysis [19].

Reflexivity was maintained throughout the analysis and writing by recording, discussing and challenging established assumptions. The first author (N.S.) conducted all interviews and focus groups and collected all log entries. She was not known to the participants and was based in the external University setting. This ensured no preconceptions in relationship to health service delivery.

Ethical approval was granted by the Research Ethics Committees Northern Ireland, Reference 16/NI/0044, IRAS project ID: 18435. The ethics committee was allocated as part of the Integrated Research Application System (IRAS) process.

## Results

Tables 1 and 2 include details about consented patients from each site. Most diagnoses were normal variants or non-serious lower limb pain conditions (Table 2). Most patients were assessed within a short waiting time (median time to first assessment 2.9 weeks; range 1–16 weeks). The majority (site 1 = 95%, site 2 = 52%) had the first appointment within 4 weeks; the longest times reflected family requests to re-book appointments (e.g. around holidays or work schedules). The differences in the time to first assessment between sites 1 and 2 probably reflect the different service set-up at the two sites (e.g. referral pathways, clinic set-ups, frequency of clinics).

**Table 1:** Details of children triaged to community paediatric physiotherapy and/or podiatry

Characteristic	Site 1	Site 2
*n*	*n* = 75 consented from a total of 101 patients triaged to the community	*n* = 48 consented from a total of 61 patients triaged to the community
Age, years	Median: 8 years	Median: 7 years
	Range: <1–15 years	Range: 1–15 years
Sex, *n* (%)	Male: 33 (44%)	Male: 22 (46%)
	Female: 42 (56%)	Female: 26 (54%)
Time to first appointment	Median: 2.7 weeks	Median: 3.7 weeks
	<2 weeks: *n* = 23/75 (31%)	<2 weeks: *n* = 11/48 (23%)
	<4 weeks: *n* = 71/75 (95%)	<4 weeks: *n* = 25/48 (52%)
Discipline	Podiatry: *n* = 38/75 (51%)	Podiatry: *n* = 31/48 (65%)
	Physiotherapy: *n* = 37/75 (49%)	Physiotherapy: *n* = 17/48 (35%)
Outcome	Discharged after first visit: *n* = 3/75 (17%)	Discharged after first visit: *n* = 11/48 (23%)
	Discharged with self re-referral option: *n* = 28/75 (37%)	Discharged with option of self re-referral: *n* = 5/48 (10%)
	On-going treatment: *n* = 26/75 (35%)	On-going treatment: *n* = 31/48 (65%)
	Onward hospital referral: *n* = 3/75 (4%)^a^	Onward hospital referral: *n* = 1/48 (2%)^b^
	Other: *n* = 5/75 (7%, data not available)	
Documented intervention(s)	Footwear/exercise advice ± orthotics: *n* = 60 (80%)	Footwear/exercise advice ± orthotics: *n* = 31 (65%)
	Explanation/reassurance alone: *n* = 15 (20%)	Explanation/reassurance alone: *n* = 16 (33%) Other (walking aid): *n* = 1 (2%)

a Suspected hip dysplasia, *n* = 1 (excluded by orthopaedics); marked hypermobility, *n* = 1; and suspected osteoid osteoma, *n* = 1 (confirmed by orthopaedics).

a Toe walking, *n* = 1, referred to paediatrics with poor coordination (subsequently confirmed to be normal gait).

**Table 2:** Diagnoses for children triaged to community paediatric physiotherapy and/or podiatry

Diagnosis	Site 1 (*n* = 75)	Site 2 (*n* = 48)
Normal variants	51 (of these, 6 had >1 normal variant)	31 (of these, 8 had > 1 normal variant)
Flat feet	27	17
In toeing	6	11
Toe walking	3	6
Curly toes	7	1
Hypermobility	8	8
Out toeing	2	0
Knock knees	4	0
Anterior knee pain	13	3
Heel pain (Sever’s disease)	3	9
Other diagnoses	8^a^	5^b^

a Leg length discrepancy, *n* = 1; suspected dysplastic hip, *n* = 1 (refuted by orthopaedics); suspected osteoma, *n* = 1 (confirmed by orthopaedics); positional talipes, *n* = 2; soft tissue strain, *n* = 1; abnormal toe nails, *n* = 1; and knee pain related to trauma, *n* = 1.

b Blistering (from footwear), *n* = 1; Kohler’s disease, *n* = 3 (osteochrondroses); and soft tissue strain, *n* = 1.

For a comparison, the routine waiting time to hospital first appointment at the time of the study was 14 weeks (source: local referral data site 1 at start of the study). The time to first hospital appointment for children triaged to hospital care was not part of our study and was not included in our ethical permission.

Many patients (46%) were discharged after the initial consultation with the community paediatric physiotherapist or podiatrist, and four patients (details in Table 1) warranted specialist opinion and were referred promptly to specialist services. The most frequent interventions by the community providers were explanation, advice on footwear/exercise and the prescription of orthotics. The proportion of children requiring on-going treatment differed between the sites and is likely to reflect a potential bias towards more complex cases being assessed at site 2.

None of the patients who had been triaged to the community re-presented to the respective NHS Trusts with a red-flag condition within 6 months of the original referral, and no patients re-referred themselves back to community services during the course of the study.

Specifically, at site 1, 398 letters of referral with MSK presentations were triaged over 6 months (∼50 referrals/month). From this total triaged, 101/398 (25%) were deemed appropriate to be assessed by community providers; 7/101 (7%) failed to attend appointments; and of the 94 attendees, 75 families consented to give feedback and for their child’s outcome data to be collected.

Site 1 and site 2 had different pre-existing services in place. In site 1, it was possible to ascertain the total number of children referred with an MSK presentation, because all GP referral letters were sent to a centralized GP e-system and received by the triage team. Before the study, all MSK paediatric referrals continued to hospital services and none was seen first in the community by paediatric physiotherapists or podiatrists. In site 2, the GP in the pre-existing service could refer direct to the paediatric physiotherapy/podiatry service based in the paediatric department at the hospital or could refer to the paediatrician, who would then assess the child first and decide whether to refer to physiotherapy or podiatry (or not). In site 2, during the course of the study, 61 referrals with MSK complaints were referred direct to paediatric physiotherapy or podiatry, and 8/61 failed to attend appointments. Most attendees (48/53) consented to data collection and providing feedback. A further 65 referrals with an MSK presentation were referred to general paediatrics, and of these, 25/65 were deemed to have been eligible for direct referral to paediatric physiotherapy/podiatry (i.e. without need for general paediatric assessment first); this judgement was based solely on the GP referral letter before their appointment, and after the assessment none was deemed to require a medical opinion from a general paediatrician.

### Perspectives from families

For those 123 consented parents of children triaged to community providers (*n* = 75 at site 1 and *n* = 48 at site 2), questionnaires were completed by 122/123, and telephone interviews were conducted with three parents (Table 3; Supplementary Table S1, available at *Rheumatology Advances in Practice* online). Family and Friends and Collaborate data showed high satisfaction scores; parents cited prompt appointments and reassurances received from the paediatric physiotherapist or podiatrist as being highly valued, with no complaints or requests for subsequent hospital specialist referral.

**Table 3:** Feedback from families: Family and Friends and Collaborate tools

Family and Friends test: recommending the service	*n* (%)	
Yes	119 (99)	
No	0	
Maybe	1 (1)	
Total *n* = 120 (2 respondents skipped this question)		

Collaborate: parent satisfaction scores (from 1 = no effort to 9 = every effort made)	Mean rating	Range
Listened to things that matter most to you about your/your child's health	8.9	8–9
Included what matters most to you in choosing what to do next	8.9	6–9
Helped understand your/your child's health issues	8.8	7–9
Total *n* = 121 (1 respondent skipped this question)		

### Perspectives from community providers (paediatric physiotherapists and podiatrists)

Feedback about the new model was generally very positive, with no concerns expressed by the parents to the providers. Furthermore, they reported that the children triaged to their care were of a similar case mix to their regular clinical practice and therefore appropriate for their existing professional skill set.

### Perspectives from triage teams

Paediatric experience was deemed really important for the triage process. At site 1, the triage team included two adult MSK nurses and an adult physiotherapist at the start of the project. It became apparent within the first focus group that they were uncomfortable and lacked confidence to triage paediatric referrals even when using the triage guide (Supplementary Table S1, available at *Rheumatology Advances in Practice* online). In contrast, the triage staff at site 1 with paediatric experience reported most triage decisions to be easy/very easy (77%), compared with 18% being easy/very easy when triage was performed by non-paediatric trained MSK triagers (Table 4). Consequently, the triage team at site 1 was changed to include HCPs with paediatric experience for the second phase at site 1, and subsequent feedback from the triagers was much more positive (Table 4). At site 2, triage was performed by a paediatric physiotherapist from the outset, with feedback that triage decisions were easy/very easy (92%).

**Table 4:** Feedback from the triage teams about the triage weekly log data from both sites

Weekly log response scale to ease of triage decision	Triage performed by adult MSK triage staff Site 1, months 1–3	Triage performed by paediatric experienced MSK triage staff Site 1, months 3–6	Triage performed by paediatric experienced MSK triage staff Site 2, months 3–9
Very easy	0	51 (46%)	2 (15%)
Easy	7 (18%)	34 (31%)	10 (77%)
Neutral	26 (68%)	18 (16%)	1 (8%)
Difficult	4 (10%)	6 (5%)	0
Very difficult	1 (3%)	2 (2%)	0
*n* (number of log entries)	38	111	13

MSK: musculoskeletal.

Triagers preferred a blended format of training, especially during initial phases of the study. At site 1, *ad hoc* informal guidance about triage decisions from doctors with a specialist MSK interest was valued. Furthermore, the two monthly quality team meetings reflecting on real data, feedback and decision-making were deemed positive and encouraged shared peer learning.

## Discussion

We have developed a new model of care as a service improvement for children, and we are not aware of similar models in child health. The RightPath model involves triage guidance to triage GP letters and identify children with MSK presentations who might be assessed and managed by community providers (paediatric physiotherapists and/or podiatrists) rather than being referred to hospital specialist services. Our study suggests that ∼25% of all children presenting to their GP with MSK presentations are appropriate for community provider assessment. We focused on the 25% for our evaluation, because these are the children who were directed away from secondary services using the triage guide; we assessed acceptability and feasibility among the parents and the providers. Our new model is timely, relevant and important, with MSK presentations in children being common [1, 2] and the need for NHS health-care services to use existing resources optimally [20].

### Strengths and limitations

Our results suggest that RightPath is feasible and acceptable (to parents, triagers and community providers); it enables prompt access to care locally and reduces variation in care. RightPath was implemented within two separate sites with different pre-existing systems in place and, although this prevented the sites from being directly comparable, we feel that these differences reflect real-life variation in primary care practice in the UK.

The model identified the right children to be assigned to a community provider for assessment. These children would all have been assessed in hospital specialist services before our study; we anticipate that many are likely to have also been referred to paediatric physiotherapy and podiatry, potentially in the community, given the high proportion of normal variants. RightPath therefore bypassed this step for a significant number of children. Given that ∼25% of patients were diverted away from secondary referral, the overall referrals to secondary care might well have reduced, but we were unable to evaluate this specifically. Furthermore, the (few) cases triaged to a community provider where concerns were subsequently raised were all fast-tracked to hospital specialist care rather than an otherwise routine elective outpatient referral had triage not taken place; hence, suggesting no delay incurred for these children. Formal validation of the triage guide and process would need more information about the diagnoses and waiting times of the children triaged to hospital care. Such information was not available in this study owing to ethical constraints but is an area for further work. The study also demonstrated high levels of satisfaction and acceptability from the parents of the children triaged to community care.

In real life, triage teams and community providers need to have more than one person on a working rota to be functional day to day. Our work was therefore to identify important components of the triage and community provider teams and how best to support them. Paediatric experience in the triage team was deemed essential, and access to a blended format for learning with case-based discussions and interprofessional learning was highly valued. No additional training for the community providers was deemed necessary, because the case mix was appropriate for their existing skill set. It is noteworthy that the proportion of children referred to community services increased over time, probably reflecting increased confidence of the triagers; it is plausible that the 25% being filtered to community care is a conservative estimate that could increase further with time.

Our methods did not influence the content of the GP referral letter. The content of the letters led to challenges to apply the triage guidance. Where possible, triage staff were asked to contact the GP to obtain further key information (e.g. duration and severity of symptoms), but this was often impractical; therefore, in the absence of information to make a decision, the triage outcome defaulted to onward referral, according to the original GP referral. We suggest that the effectiveness of triage could be improved further with a prompting tool or referral proforma to ensure that the right information is included within the GP letter to apply the triage guidance; this would also enable quantitative data description of missing data to inform feedback and in-house training.

Our study was not a formal clinical trial and not powered to address the ability not to miss serious red-flag MSK pathology (such as malignancy or infection). The 6 month follow-up window to identify any patients who re-presented into the health-care system reassuringly identified no red-flag conditions, and no patients self-referred back to the community services. We did not interview hospital-based specialist providers about the impact of our model, but we did not receive adverse feedback once the new model was operational. Notably, there were no anecdotal reports of patients who had been delayed in their referral to specialist care or reports of de-stabilization of services.

The study focused on implementation rather than cost effectiveness. The term cost needs to consider the anxiety incurred without a diagnosis, time off from work or school, and travel costs to attend hospitals and the distances involved. UK hospital-based specialist children’s services are centrally funded from NHS England; therefore, any current and potential future cost savings are reliant on the ability of local providers to release funding from central budgets. Undoubtedly, there are potential workforce implications (that require funding and resource) to accommodate a 25% increase in referrals to community providers.

More work is needed to evaluate implementation for a longer period of time across more centres. How this can be done given the different set-up of primary care services around the UK is challenging and would need consideration for trial design and to consider control for the intervention.

### Comparison with existing literature

GPs are often the first to assess patients, and many report low confidence in their ability to perform MSK assessment in children [21], probably because many primary care training schemes do not include paediatrics [22] or MSK medicine [23], despite GP learning needs being known [24]. We did not explore further the rationale for the referrals from the GP perspective, because we had no direct contact with the referring GPs, and our intervention started after the GP referral process had been initiated. There might be several factors impacting the GP referral decision-making process and, although the literature suggests lack of training and/or confidence as major factors [21–24], this is clearly an area for future study.

The need to increase exposure to paediatrics in GP training has been recognized in the UK [25]. The training for paediatric physiotherapists [26] and podiatrists [27] does, however, include competencies to identify and manage normal variants and also to identify serious MSK pathology and when to refer. Such professionals are therefore highly suitable to work with GPs to implement our model nearer to home for families and with better interface working between hospital specialist services and primary care.

### Implications for research and practice

Our study reflects evidence-based approaches for change [28] and reflects the benefits of MSK triage for adults in primary care [29]. Essentially, key features are as follows. The project team included primary care, community and specialist providers to ensure that the triage guide was fit for purpose. It was developed from an existing adult MSK triage model tailored to reflect common clinical scenarios and with full recognition that children are not little adults. Mixed methods captured real life in two sites and reflected current variation in care pathways and service delivery. Stakeholder engagement events encouraged buy in. The monthly e-Newsletter with project updates facilitated ongoing engagement (examples are available at www.rightpath.solutions). Paediatric MSK experience is integral to the triage decision-making process; our methodology allowed this key modification to the model to be made during the study. Triage teams preferred a blended learning [30] format, with shared peer learning, and PMM (www.pmmonline.org) as an adjunct resource. Key messages for implementation are given (Table 5). The triage guidance is available with Common License (www.RightPath.solutions) and has informed NICE guidance (https://cks.nice.org.uk/developmental-rheumatology-in-children#!topicSummary).

**Table 5:** Key points for successful implementation

Local agreement that self-limiting and normal variant MSK conditions should be seen in the community by suitably trained physiotherapists and podiatrists. Agreement and support of service funders to develop and implement the model and to ensure that community-based staff have the capacity and capability to facilitate triage and prompt, accurate assessment. The project team should include representatives from all local stakeholders, and a wider stakeholder engagement event is advised to engage, gain support (buy in) and air concerns. Triage staff should have paediatric experience and be familiar with normal MSK development and normal variants, with rapid access to experienced clinical support. Access to informal *ad hoc* specialist support and advice for triage staff is key, especially within the early stages of implementation. Triage teams are needed with more than one person on a working rota, in order to be functional day to day. Training of triage staff should include case-based discussions and anonymized real-life scenarios to practise use of the triage guide. Training in referral software and local pathways is needed in order that all triage staff manage referrals in a consistent manner. Interprofessional learning within multidisciplinary teams and on-going training and support are vital to maintain the quality of triage. Audit systems are required to capture activity and inform service redesign, including patient/parent outcomes in the service, with regular feedback to clinical staff.

MSK: musculoskeletal.

Our model aims to facilitate the right care first time and to reduce variation in care. Our study has shown better clarity of care pathways and improved interface working to identify correctly those who can be managed by community providers. There is likely benefit to the NHS, clinicians, children and families, with improved cross-boundary working and better utilization of resources. More work can be done on cost effectiveness, safety and implementation on a wider scale. There is a need to explore ways to support GPs with the referral process (e.g. with referral proformas and training), expand the role of allied health professionals in the community and explore the impact on waiting times and the capacity of hospital specialist services. RightPath might also have transferable value to other areas of child health.

## Supplementary Material

rkaa057_Supplementary_Data

## References

[1] Tan A Strauss VY Protheroe J Dunn KM. Epidemiology of paediatric presentations with musculoskeletal problems in primary care. BMC Musculoskelet Disord 2018;19:40. 29409492 10.1186/s12891-018-1952-7 PMC5801684

[2] Prathivadi Bhayankaram N Lacey RJ Barnett LA Jordan KP Dunn KM. Musculoskeletal consultations from childhood to adulthood: a longitudinal study. J Public Health 2019;28:1–7. 10.1093/pubmed/fdz141 31774535

[3] Foster HE Eltringham MS Kay LJ et al Delay in access to appropriate care for children presenting with musculoskeletal symptoms and ultimately diagnosed with juvenile idiopathic arthritis. Arthritis Rheum 2007;57:921–7. 17665486 10.1002/art.22882

[4] Saxena S Francis N Sharland M. Primary care of children: the unique role of GPs. Br J Gen Pract 2012;62:340–1. 22781967 10.3399/bjgp12X652166 PMC3381241

[5] Yeo A James K Ramachandran M. Normal lower limb variants in children. BMJ 2015;350:h3394. 26152216 10.1136/bmj.h3394

[6] Ciafaloni E Fox DJ Pandya S et al Delayed diagnosis in Duchenne muscular dystrophy: data from the Muscular Dystrophy Surveillance, Tracking, and Research Network (MD STARnet). J Pediatr 2009;155:380–5. 19394035 10.1016/j.jpeds.2009.02.007 PMC5884059

[7] Craft A. Are health services in England failing our children? BMJ 2007;335:268–9. 17656502 10.1136/bmj.39282.492801.80 PMC1941870

[8] Dang-Tan T Trottier H Mery LS et al Delays in diagnosis and treatment among children and adolescents with cancer in Canada. Pediatr Blood Cancer 2008;51:468–74. 18454472 10.1002/pbc.21600

[9] Foster HE Cabral DA. Is musculoskeletal history and examination so different in paediatrics? Best Pract Res Clin Rheumatol 2006;20:241–62. 16546055 10.1016/j.berh.2005.11.001

[10] Kocher MS Bishop JA Weed B et al Delay in diagnosis of slipped capital femoral epiphysis. Pediatrics 2004;113:e322–5. 15060261 10.1542/peds.113.4.e322

[11] McErlane F Foster HE Carrasco R et al Trends in paediatric rheumatology referral times and disease activity indices over a ten-year period among children and young people with Juvenile Idiopathic Arthritis: results from the childhood arthritis prospective Study. Rheumatology (Oxford) 2016;55:1225–34. 27016664 10.1093/rheumatology/kew021 PMC4911538

[12] Smith EMD Foster HE Gray WK et al Predictors of access to care in juvenile systemic lupus erythematosus: evidence from the UK JSLE Cohort Study. Rheumatology (Oxford) 2014;53:557–61. 24310297 10.1093/rheumatology/ket402

[13] Rotter T Kinsman L Machotta A et al Clinical pathways for primary care: effects on professional practice, patient outcomes, and costs. Cochrane Database of Syst Rev 2013;8:1–12. 10.1002/14651858.CD006632.pub2 20238347

[14] Oldham Council. Oldham Public Health Annual Report. 2017 https://www.oldham.gov.uk/downloads/file/5240/public_health_report_for_oldham (October 2019, date last accessed).

[15] Office for National Statistics. Local Authority Profile. 2018 https://www.nomisweb.co.uk/reports/lmp/la/1946157067/report.aspx (October 2019, date last accessed).

[16] Smith N Rapley T Jandial S et al Paediatric musculoskeletal matters (pmm) – collaborative development of an online evidence based interactive learning tool and information resource for education in paediatric musculoskeletal medicine. Pediatr Rheumatol Online J 2016;14:1. 26728031 10.1186/s12969-015-0062-4 PMC4700751

[17] Coulter A. Patient feedback for quality improvement in general practice. BMJ 2016;352:i913. 26893467 10.1136/bmj.i913

[18] Elwyn G Barr PJ Grande SW et al Developing CollaboRATE: a fast and frugal patient-reported measure of shared decision making in clinical encounters. Patient Educ Couns 2013;93:102–7. 23768763 10.1016/j.pec.2013.05.009

[19] Rapley T. Some pragmatics of qualitative data analysis In Silverman D. ed. Qualitative research: issues of theory, method and practice. Vol. 3 London: Sage, 2011: 273–90.

[20] NHS England. Musculoskeletal conditions. 2019 https://www.england.nhs.uk/ourwork/clinical-policy/ltc/our-work-on-long-term-conditions/musculoskeletal/ (October 2019, date last accessed).

[21] Jandial S Myers A Wise E Foster HE. Doctors likely to encounter children with musculoskeletal complaints have low confidence in their clinical skills. J Pediatr 2009;154:267–71. 18823907 10.1016/j.jpeds.2008.08.013

[22] Modi N Simon C. Child health care: adequate training for all UK GPs is long overdue. Br J Gen Pract 2016;66:228–9. 27127270 10.3399/bjgp16X684853 PMC4838420

[23] Wise EM Walker DJ Coady DA. Musculoskeletal education in general practice: a questionnaire survey. Clin Rheumatol 2014;33:989–94. 24510025 10.1007/s10067-013-2479-9

[24] Goff I Boyd DJ Wise EM Jandial S Foster HE. Paediatric musculoskeletal learning needs for general practice trainees: achieving an expert consensus. Educ Prim Care 2014;25:249–56. 25625831 10.1080/14739879.2014.11494290

[25] Stiris T del Torso S Mercier J-C et al Improving paediatric care in the community. Lancet 2015;385:1505. 10.1016/S0140-6736(15)60745-0 25933273

[26] Burslem J McAtasney D McGarrity K et al Working with children - guidance on good practice. Chartered Society of Physiotherapy 2016 https://apcp.csp.org.uk/system/files/working_with_children_2016_1.pdf (October 2019, date last accessed).

[27] Williams CM Nester C Morrison SC. International approaches to paediatric podiatry curricula: it’s the same, but different. J Foot Ankle Res 2019;12:28. 31086569 10.1186/s13047-019-0339-9 PMC6507174

[28] Murray E Treweek S Pope C et al Normalisation process theory: a framework for developing, evaluating and implementing complex interventions. BMC Med 2010;8:63. 20961442 10.1186/1741-7015-8-63 PMC2978112

[29] Joseph C Morrissey D Abdur-Rahman M Hussenbux A Barton C. Musculoskeletal triage: a mixed methods study, intergrating systematic review with expert and patient perspectives. Physiotherapy 2014;100:277–89. 25242531 10.1016/j.physio.2014.03.007

[30] Graham CR. Blended learning systems In Bonk CJ Graham CR Cross J Moore MG. eds. The handbook of blended learning: global perspectives, local designs. San Francisco, CA: Pfeiffer Publishing, 2006: 3–21.

