# Supplementary material for: RightPath: a model of community-based musculoskeletal care for children
Source: Rheumatol Adv Pract. 2020 Oct 16;4(2):rkaa057. doi: 10.1093/rap/rkaa057 (PMC7661842; doi:10.1093/rap/rkaa057)
Supplement: rkaa057_Supplementary_Data [file rkaa057_supplementary_data.docx]

**Supplementary Table S1. Perspectives from families, triage teams and community providers**

| **Perspectives from families** | |
| --- | --- |
| **1. ‘Child focused personable service’** | |
| Families reported service providers had appropriate experience. | “Lovely treatment and service today. She [physiotherapist] was lovely and patient with my little boy. Very understanding especially as he wanted to play rather than get examined”.  “Absolutely brilliant. [Physiotherapist] helped my 12 year old to understand what he had”.  “Friendly, approachable, tried to explain in layman terms”. |
| **2. Suitability of service** | |
| Families reported being listened to, given appropriate advice & support. | “Excellent; time was spent talking about the problem & then a thorough examination was done”.  “Very good. They made sure I understood everything and what to do next if I had any other issues”. |
| Families liked that referral to service was quick and with no need for re-referral through the GP. | “Appointment was quick and efficient, reviewing the issue and solution thoroughly”.  “A solution was achieved to relieve pain. A follow-up appointment was made there and then”.  “The treatment received today was very good! It exceeded my expectations as was very friendly, listened to me and made me feel comfortable”. |
| Preferred community environment. | “I much preferred being seen in the community rather than a hospital environment”  “Originally I wanted to see really a specialist but by the end of the consultation with the physio I felt like it wasn’t necessary and he really, yeah he went through all the tests and was very thorough and he identified the problems and what we had to work on from a physical point of view”.  “I think parents feel that it's good because they're seen locally within their own community”. |
| **Perspectives from triage teams** | |
| **1. Paediatric musculoskeletal experience to inform triage decisions** | |
| Referral guidance not a substitute for clinical experience.  Concerns about personal paediatric experience in the decision making process. | Adult musculoskeletal nurses: “At times challenging, but I think that’s because we’re not paediatric trained, rather than the processes”.“I was just apprehensive because it was children”. “It just feels like a huge responsibility when it’s children for some reason”.  Podiatrist/Physiotherapist with paediatric expertise: “I think because paediatrics is very, very different to the adult work you see things that you just would never come across in the adult world, certainly, and there are lots of different conditions that are very specific to Paediatrics that you just don’t ever see or that you wouldn’t ever come across as an adult clinician. |
| **Perspectives from triage teams: training and support** | |
| **1. Blended learning approach** | |
| Sample triage letters, case discussion and facilitated group sessions.  PMM as a ‘go-to’ e-resource. | “Because you can go through the triage guide, but actually it’s not until you sit there with a letter and try and make it make sense”.  “I think that was the most useful training thing, the triage guide but then in conjunction with the discussion of letters”.  “That was good that, going through the case studies as examples. It gives you an idea”.  “We sort of discussed it between each other, which was useful because otherwise I think I would have felt a bit isolated with it”.  “I like it [PMM]. I’ve used it a lot over the last year or so. I suppose I started it before this study. I found it really good, yes, especially just for knowing what’s normal and the normal variants and being confident then to say to parents, ‘look that’s just a normal variant’”.  “I found it [PMM] good. I refer back to it a lot, a good bit if I'm not sure of something”.  “Even as a training tool for physios coming into paediatric orthopaedics as case stuff, I think PMM is a good resource if you're not too sure. It's a good learning tool”. |
| **Perspectives from community providers** | |
| **1. ‘No different to our everyday practice’** | |
| Clinical caseload appropriate for their skill set. | “We do this work day in, day out. We’ve been doing it for 15 years, so it’s what we would expect to come through our doors”.  “I suppose with the patients that come through to us, because the conditions are what we do routinely anyway, it’s very easy for us to explain what’s going on. We’re used to that. That’s one of our skills, I suppose, understanding how to discuss it with the kid, as well as with the parent”.  “The majority of the ones we see are, what we'd class as, normal walking variants anyway, so things like in-toeing and hypermobility and flat feet. Yes, there's the odd one that you think might be a bit more complicated than that, but generally I would say it was business as usual for us”.  “There have been no problems. The parents, as well as the children, have been happy with the consultation and the treatment plans, and I think, although they might have been initially told by the GP that they were going to be seen by a consultant, or the orthopaedic team, they didn’t really expect that, as such. Nobody said, ‘Oh, well, I thought I was seeing a consultant. How come I’ve come here when I should have been going to the hospital’”. |
| **2. Workable process** | |
| 'RightPath' as a model of care is a positive change and better for families. | “It’s one of those things, in theory where you wonder why it’s not in place anyway. Good triage at the source should make everything work a little bit more efficiently”.  “We do know that children are being referred, like we've said, in duplicate to different services, or they're being referred to a paediatrician when they don't need to be. So I do think it's a good idea. So if we can get the children to the right services then that would be better for everybody.” |

GP: general practitioner; PMM: paediatric musculoskeletal matters ([www.pmmonline.org](http://www.pmmonline.org))
